# Applying a deterrence nudge strategy for promoting stair usage in a university setting

**DOI:** 10.1186/s12889-024-19592-6

**Published:** 2024-08-13

**Authors:** Chung Gun Lee, Jiyeon Chu, Ruoqi Mao, Hansol Kim, Eun-Young Lee, Seiyeong Park, Taeeung Kim

**Affiliations:** 1 Department of Physical Education, College of Education. 71-1, Seoul National University, Seoul, 08826 Republic of Korea; 2 Institute of Sport Science, Seoul National University, Seoul, 08826 Republic of Korea; 3 Department of Gender Studies, School of Kinesiology and Health Studies, Queen’s University, Kingston, ON Canada; 4 School of Kinesiology and Health Studies, Queen’s University, Kingston, ON Canada

**Keywords:** Deterrence nudge, Point-of-decision prompts, Time-targeted message, Stair use, University setting

## Abstract

**Purpose:**

This study aimed to examine whether indirectly deterring elevator use through time-targeted Point-of-Decision Prompts (PODPs) efficiently increased stair usage in a university setting.

**Methods:**

A quasi-experimental design (pre-post design) was employed over 2 weeks in September 2023. Baseline observations were conducted for 1 week prior to signage placement at two locations. The intervention in this study lasted for 1 week, immediately following baseline observations. Three hundred and thirty-one and 384 participants were observed during the baseline and intervention periods, respectively. Logistic regression analysis was used to examine the increase in the act of ascending the stairs.

**Results:**

Our intervention, which focused on time-related messages, effectively increased stair usage among university students (coefficient = 0.435, *p*-value < 0.01). Furthermore, females (coefficient = -0.820, *p*-value < 0.05) and individuals aged ≥ 30 years (coefficient = 1.048, *p*-value < 0.01) were notably more likely to be influenced by our intervention than males and individuals aged < 30 years.

**Conclusion:**

Indirectly discouraging elevator use through time-targeted PODPs may amplify the effects of the previously employed time-related messages. Our findings suggested that a deterrence nudge should primarily be directed towards promoting stair usage among females or individuals aged ≥ 30 years.

## Introduction

Engaging in moderate to vigorous physical activity can help achieve important health benefits, such as reducing the risks of obesity, diabetes, osteoarthritis, certain types of cancer, and cardiovascular diseases [1]. Also, evidence suggests that regular physical activity helps alleviate depressive symptoms and anxiety [2]. Despite extensive evidence of several health benefits associated with physical activity, only one in five (20.8%) university students in South Korea participate in the recommended level of physical activity [3]. This rate is considerably lower than that of students in the United States, where approximately half of university students meet the recommended level of physical activity [4].

Low levels of physical activity among South Korean university students can be partly attributed to societal and environmental factors related to lower daily energy expenditure, due in part to an increase in energy-saving devices, greater use of public automated transport systems, and a reduction in occupational physical activity [5, 6]. In addition, a strong emphasis on education as a means to achieve success in South Korea places a considerable academic burden on university students. South Korean university students frequently have busy schedules; they take multiple courses, assignments, and exams. As a result, there might be insufficient time to perform physical activities [7]. One of the possible strategies to promote physical activity among South Korean university students is the accumulation of small bouts of physical activity, such as taking the stairs instead of the elevator, throughout the day.

Promoting stair usage is recognized as one of the most effective strategies to increase physical activity at the population level [8]. Unlike structured exercise or sports, stair use does not require specialized sports equipment and gear. It is a low-cost and easily accessible activity for individuals in their daily lives. Stair climbing requires energy expenditure approximately nine times greater than the resting state, and fulfills the minimum intensity of physical activity required for various health benefits [9–11]. Climbing stairs has been shown to improve muscle strength [12], body composition [13], and maximal aerobic capacity [14]. It has been shown to reduce low-density lipoprotein cholesterol [15, 16]. For these reasons, stair use has been recommended as one of the activities of daily living to improve health [17], and can help people achieve the recommended levels of physical activity [18].

Recent systematic reviews have shown that point-of-decision prompts (PODPs) effectively increase the likelihood of stair usage instead of elevators or escalators among various populations, including university students [19–21]. PODPs are signs placed near elevators, escalators, or stairwells that encourage people to use the stairs. Jennings et al. not only provided an undated review regarding various interventions to increase stair usage, but also explored differences in intervention components that may affect effectiveness of stair use interventions [19]. According to Jennings et al., [19] the content of signs used in PODP interventions can be divided into six categories: (1) health (use stairs to promote health); (2) energy expenditure (use stairs to burn calories); (3) weight (use stairs for weight loss); (4) fitness (use stairs to increase fitness); (5) time (use stairs to save time); and (6) others. Although a few PODP interventions for increasing stair usage included time-related messages in their signs, PODP interventions targeting time often reported more effectiveness (88%; 15 out of 17 studies reported effectiveness) than those targeting other categories, such as health (78%; 49 out of 63 studies reported effectiveness) or energy expenditure (69%; 25 out of 36 studies reported effectiveness). Moreover, to the best of our knowledge, only two studies have used PODPs with time-related messages to increase stair usage in university settings; the results of these two studies have been inconsistent [22, 23]. Therefore, more research is required to examine the impact of stair-use promotional signage using PODPs with time-related messages in university settings. This is important because university students and staff are mostly focused on moving from one place to another in a timely manner [19].

PODPs are typically designed to modify a certain health behavior by displaying information about healthier choices or establishing deterrence to relatively unhealthy choices (e.g., "Please limit elevator usage to those who are unable to use the stairs."). However, current PODP studies for promoting stair usage have mostly focused on promoting stair-use behavior, rather than deterring elevator or escalator use [21]. To our knowledge, there was only one PODP study that used a deterrent sign to limit elevator usage [21]. This was, in part, because restricting the use of elevators can potentially lead to dissatisfaction and complaints among people in that building. Nevertheless, because PODPs aiming to reduce the perceived attractiveness and accessibility of elevators are likely to exhibit promising potential as effective environmental interventions, interventions aimed at promoting stair usage should consider the indirect utilization of a deterrent message.

"Deterrence nudge" refers to the concept of employing indirect methods to prevent or inhibit certain behaviors or events without involving direct pressure or threat [24]. Instead, it involves efforts to induce changes in people's behavior through social, economic, or cultural influences [25]. For instance, creating a cultural and social environment where alcohol consumption is deemed socially unacceptable aims to discourage such behavior. These cultural changes can play a role in modifying behavior without relying on direct punishment or sanctions. Since individuals in university settings typically know each other through work, classes, and various activities, inducing behavioral changes through social, economic, or cultural influences using a deterrence nudge strategy may be highly effective within this population. Therefore, the objective of this study was to investigate whether indirectly deterring elevator use through time-targeted PODPs effectively increases stair usage among South Korean university students.

## Methods

### Setting and design

A quasi-experimental design was employed over 2 weeks in September 2023. Baseline observations were conducted for 1 week (weekdays only) prior to signage placement at two locations. The intervention in this study lasted for 1 week (weekdays only), immediately following the baseline observations. All observations were conducted from 11 AM to 1 PM, when target individuals were most likely present. This study focused exclusively on the act of ascending the stairs. The intervention was conducted at a university building in Seoul, South Korea. No special events were observed in the building during the experimental period. The building comprised five stories, with one main stairwell and two adjacent elevators. The stairwell was visible from the elevators, clean, painted, adequately ventilated, and well-lit. Notably, the elevators operated at a slower pace than usual. All observations were conducted on the first floor, where the main entrance to the building was located. Two signs were strategically positioned within the decision-point area adjacent to the stairwell and elevators, and were displayed solely on the first floor, consistent with the methodologies employed in previous studies [26, 27]. Ethical approval for this study was obtained from the Institutional Review Board at Seoul National University.

### Intervention

**Fig. 1:**
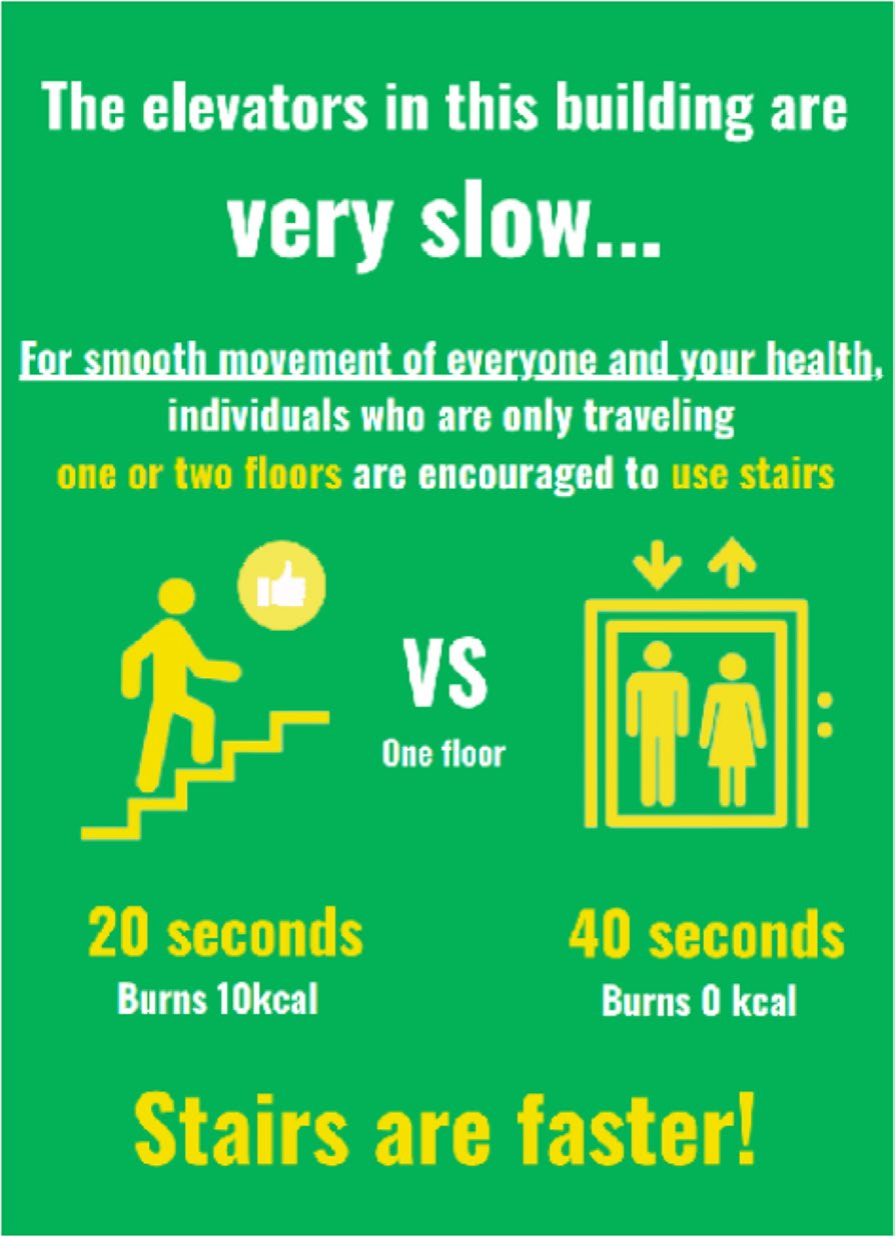
The English version of the actual poster used for increasing stair use instead of taking elevators

The same two posters were used with the following messages: (1) “The elevators in this building are very slow. For smooth movement of everyone, individuals who are only traveling one or two floors are encouraged to use stairs.” (indirectly deterring elevator use through a time-targeted message), (2) “Taking the elevator to ascend one floor takes 40 s (including waiting time), whereas using the stairs takes 20 s.” (time-targeted message), (3) “Use stairs for your health.” (health-targeted message), and (4) “Using the stairs to go up one floor burns approximately 10 kilocalories, while taking elevator burns 0 cal” (energy expenditure-targeted message). The first message was the main message, whereas the other three were additional messages. These two A1-sized posters were fastened for ease of use and positioned on the first floor of the building. The posters used in this study are shown in Fig. 1.

### Measures

Three trained graduate students recorded the acts of ascending the stairs and using the elevators, along with demographic characteristics such as externally perceived sex (male and female) and observed age (≥ 30 and < 30 years). Individuals carrying heavy or bulky packages were excluded [28, 29]. Once trained, each graduate student was repeatedly assessed for reliability throughout the coding process. The inter-observer reliability exceeded 0.90 for all measures.

### Statistical analysis

Logistic regression analysis was used to examine the increase in the act of ascending the stairs. The dependent variable was the choice between using either the stairs or the elevators. The independent variables included the following: externally perceived sex (male and female), observed age (≥ 30 and < 30 years), and intervention (baseline and intervention periods). Interaction terms were included in the model to analyze how various subgroups (sex and observed age) interacted with the intervention, particularly when influencing an individual’s decision to use either the stairs or the elevators. All analyses were performed using SAS version 9.4 (SAS Institute Inc., Cary, NC, USA).

## Results

The target population included all the individuals entering the building. During the baseline and intervention periods, 331 and 384 participants were observed, respectively. In both periods, males were slightly more prevalent than females; most individuals were < 30 years old. Stair usage rate was significantly higher during the intervention period than during the baseline period. During the baseline period, > 60% of the participants used the stairs instead of the elevators, whereas > 70% opted for stairs during the intervention period. Characteristics of the observed individuals are presented in Table 1.

**Table 1:** Characteristics of observed population by study period

	Baseline N (%)	Intervention N (%)
Sex		
Males	185 (55.89)	242 (63.02)
Females	146 (44.11)	142 (36.98)
Observed age		
30 or more	66 (19.94)	102 (26.56)
Less than 30	265 (80.06)	282 (73.44)
Target behavior		
Stair use	202 (61.03)	270 (70.31)
Elevator use	129 (38.97)	114 (29.69)
Total	331 (100.00)	384 (100.00)

**Fig. 2:**
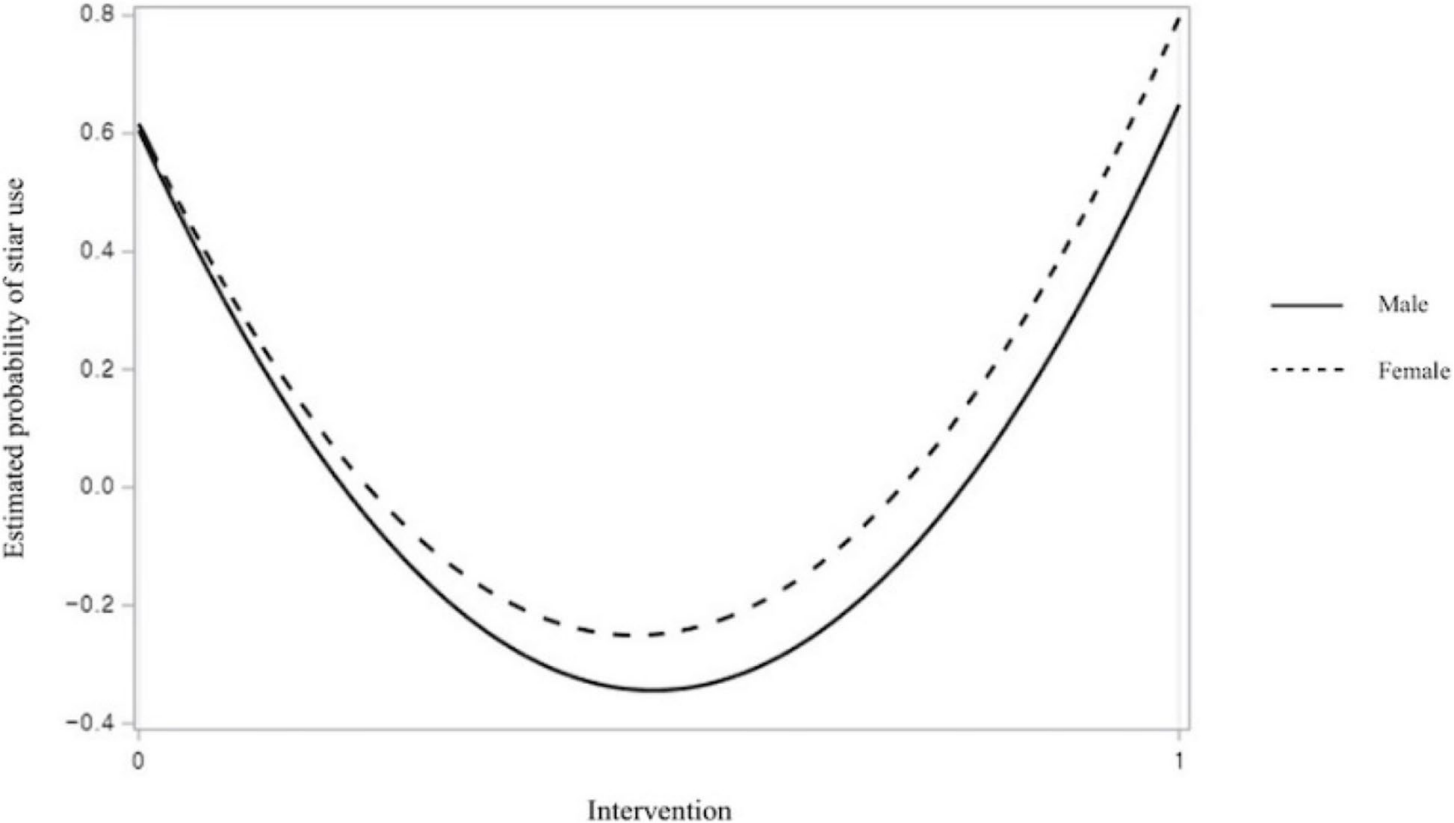
The effect of interaction between sex and intervention on stair use

**Fig. 3:**
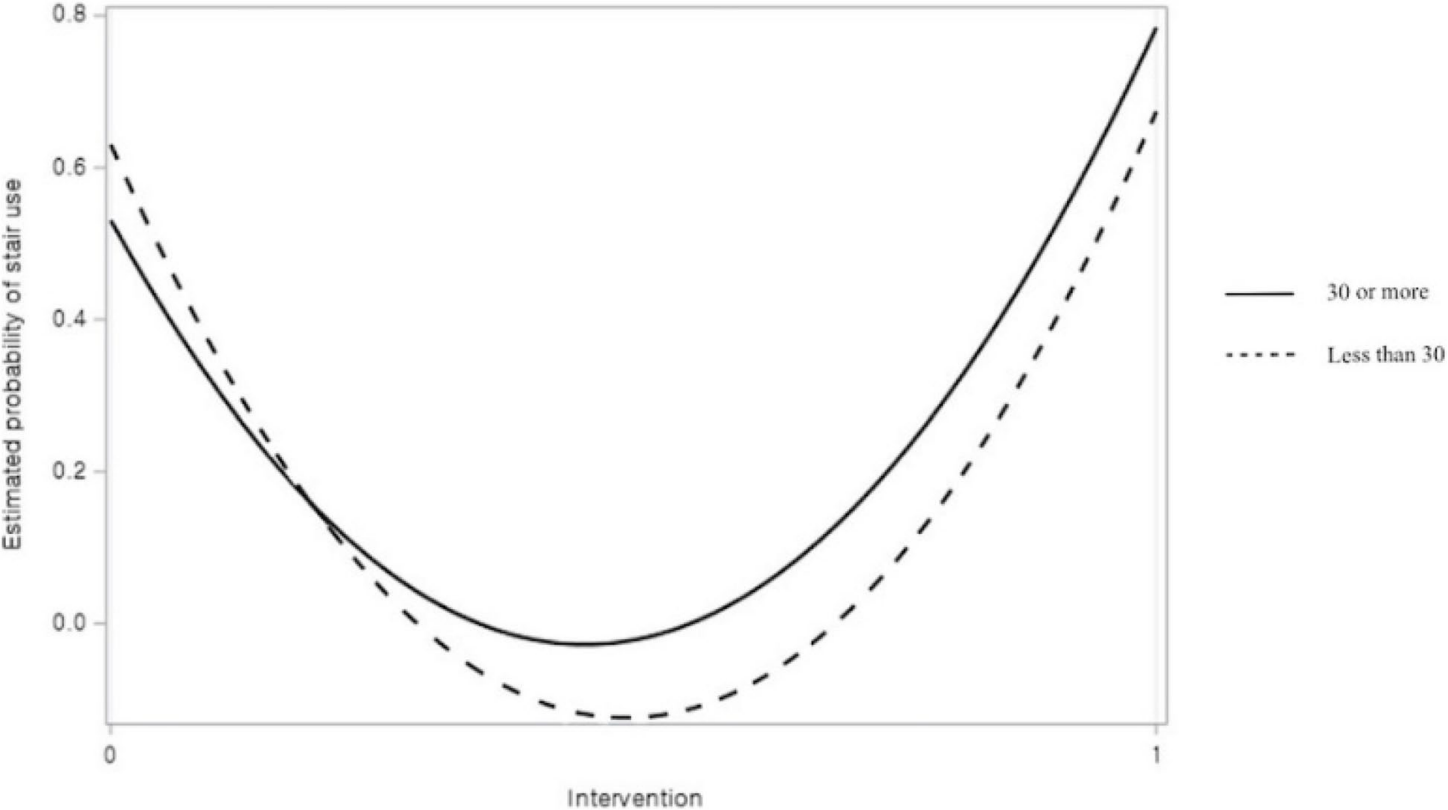
The effect of interaction between observed age and intervention on stair use

The results of the multiple logistic regression analysis concerning the use of stairs, as opposed to elevators, are presented in Table 2. In Model 1, males were less likely to use stairs than females (*p* < 0.05). Participants exhibited a significantly higher probability of using the stairs during the intervention period than during the baseline period (*p* < 0.01). Observed age did not significantly affect stair usage. In Model 2, two interaction terms were additionally included from Model 1. Females were more susceptible to the intervention than males (*p* < 0.05, see Fig. 2). Moreover, individuals aged ≥ 30 years were more likely to use the stairs during the intervention period than those aged < 30 years (*p* < 0.01, see Fig. 3).

**Table 2:** Multiple logistic regression of using stairs instead of elevators (*N* = 715)

Independent variables	Model 1 Coef (SE)	Model 2 Coef (SE)
Intercept	0.640 (0.150)**	0.516 (0.173)
Sex		
Males	-0.396 (0.167)*	0.034 (0.235)
Females (ref)		
Observed age		
30 or more	0.172 (0.193)	-0.421 (0.286)
Less than 30 (ref)		
Intervention		
Baseline period (ref)		
Intervention period	0.435 (0.160)**	0.719 (0.275)**
Interactions		
Sex * Intervention		-0.820 (0.343)*
Observed age * Intervention		1.048 (0.397)**
-2LL	903.678	892.444

*Coef* Coefficient, *SE* Standard error, *ref* Reference category, *-2LL* -2 log likelihood

^*^p < .05

^**^p < .01

## Discussion

This study examined whether indirectly deterring elevator usage through PODPs increased stair usage among university students at a single university in South Korea. Among the various types of content used in PODP interventions promoting stair usage in previous intervention studies, our study primarily focused on time-related messages; only a few PODP studies have concentrated on this aspect despite their relatively stronger effectiveness as compared to other intervention strategies. Consequently, our PODP intervention, which focused on time-related messages, effectively increased stair usage among individuals entering the university building. Furthermore, females and individuals aged ≥ 30 years were notably more likely to be influenced by our intervention than males and individuals aged < 30 years.

Our PODP intervention increased the stair usage rate from 61.52% to 69.41%, which is an increase of approximately 8%. Previous PODP interventions focusing on time-related messages to promote stair usage resulted in an increase from -0.6% to 9%, wherein the mean increase was approximately 4.65% [22, 30–34]; only one study demonstrated an increase of > 8% in the stair usage rate [34]. Our intervention can be considered highly effective, given that the intervention duration was relatively short (1 week) and that the baseline stair usage rate was considerably high (61.52%) as compared to other studies. In previous studies, the mean stair usage rate during the baseline period was 17.62%; the mean duration of the intervention was 3.11 weeks [22, 30–34]. It may be possible that indirectly deterring elevator use through time-targeted PODPs may potentially have amplified the impact of time-related messages used in previous studies. Our results suggested that deterrent nudges were effective not only for taxpayers, but also for elevator users [24].

Furthermore, our PODP intervention proved to be more effective for females and individuals aged ≥ 30 years than for males and individuals aged < 30 years. Specifically, our PODP intervention increased the stair usage rate among females from 61.64% to 79.58%, reflecting an increase of approximately 18%. This was a noteworthy result; three previous studies using PODPs with time-related messages also demonstrated a higher increase in stair usage rates among females than males, which was approximately from 6 to 12% [23, 27, 32, 33]. According to Eagly [35], females tended to exhibit prosocial behavior in close relationships, whereas males were more likely to engage in such behavior towards strangers and in support of social collectives. Indirectly deterring elevator use with the message, "The elevators in this building are very slow. For the smooth movement of everyone, individuals traveling only on one or two floors are encouraged to use the stairs.", may have triggered the females' prosocial behavior in our study; people tend to meet more regularly and have closer relationships in a university setting than in other settings, such as in train stations or shopping centers. Furthermore, when there was a perceived social norm that values health or environmental considerations, females were more likely to conform to these norms, as they can be more sensitive to social cues [36]. Also, though evidence was limited to a specific context, females, in general, may be more health-conscious and, therefore, may have been more receptive to our health-related messages [37]. However, in this study, the investigators determined the participants' sexuality based on the appearance or behavior of the subjects, so caution is needed in interpreting the results of this study.

There was an even more pronounced increase in the stair usage rate among individuals aged ≥ 30 years than among those aged < 30 years during our PODP intervention. The stair usage rate for individuals aged ≥ 30 years increased from 53.03% to 78.43% (a 25% increase). Previous studies on the prosocial behaviors of older individuals suggested that prosocial actions were motivated by a genuine altruistic concern for the well-being of other people or the common good; this concern tends to increase with age [38]. For this reason, individuals aged ≥ 30 years may have been more influenced by our PODP, which recommended using the stairs to facilitate smoother movement of other people in the building, as compared to those aged < 30 years. It was also possible that older individuals in this study may have been influenced by the health-targeted (i.e., “use stairs for your health.”) or energy expenditure-targeted (i.e., “using the stairs to go up one floor burns approximately 10 kilocalories, while taking elevator burns 0 cal.”) messages in our PODP. Older people were more susceptible to age-related issues and have a higher likelihood of manifesting chronic diseases [39]. However, since the participants in this study were relatively young population (mostly undergraduate and graduate students), health-related message may not have had a substantial impact on them. Again, caution needed when interpreting these results because participants' age is determined by investigators through merely looking at their behavior and appearance. Again, caution is warranted in interpreting these results as investigators determined participants' age solely through observations of their behavior and appearance.

This study had several limitations. First, all observations were confined to the first floor, where the main entrance of the building was located, thus making it impossible to determine the actual number of flights made by the participants. However, like most other PODP studies promoting stair usage, this approach was adopted because of the limited methodologies and resources. Second, the intervention period lasted for only 1 week. The short duration of the intervention implies that no conclusive remarks can be drawn about its long-term effects. Third, no follow-up investigations were conducted after the intervention. Future studies should include follow-up assessments to determine whether the observed changes persisted over an extended period. Fourth, since age and sex were recorded based on the investigators' judgement, they might have influenced the study results. However, in accordance with previous studies, our investigators were consistently assessed for reliability throughout the coding process, and pilot data also indicated reliable observations among both over and under 30 years of age. Fifth, since we included four different types of messages on the same poster without comparing them to control posters containing only one message each, it is not possible to determine which message discouraged elevator use and/or promoted stair use definitively. Sixth, the participants in this study are limited to South Korean university students or staff. The generalizability of our results should be considered when applying them to other populations. Finally, because our study did not include a control group comprising similar building types and users, it was possible that other factors may have influenced the stair usage among the participants. Despite these limitations, our PODP intervention, focusing on time-related messages, can be deemed highly effective as compared with other similar PODP interventions. It is plausible that indirectly discouraging elevator use through time-targeted PODPs may amplify the impact of previously employed time-related messages. Our findings also underscored that this intervention was particularly effective for females and individuals aged ≥ 30 years, thereby suggesting that a deterrent nudge should primarily be directed towards promoting stair usage among these populations.

## Data Availability

The datasets generated and/or analyzed in this study are available from the corresponding author on reasonable request.
